# Surgical and Functional Outcome after Endoprosthetic Reconstruction in Patients with Osteosarcoma of the Humerus

**DOI:** 10.1038/s41598-018-34397-5

**Published:** 2018-11-08

**Authors:** Christoph Böhler, Stephan Brönimann, Alexandra Kaider, Stephan E. Puchner, Irene K. Sigmund, Reinhard Windhager, Philipp T. Funovics

**Affiliations:** 1 Department of Orthopaedic Surgery, Medical University of Vienna, Waehringer Guertel 18-20, 1090 Vienna, Austria; 2 Center for Medical Statistics, Informatics, and Intelligent Systems - Section for Clinical Biometrics, Medical University of Vienna, Waehringer Guertel 18-20, 1090 Vienna, Austria

## Abstract

Endoprosthetic reconstruction (EPR) is the most widely used reconstruction technique after humeral osteosarcoma (OSA). Complications are common and function is often compromised due to the premise of wide resection. In the current study we evaluated (1) the risk of complications after resection and EPR; (2) the functional outcome and how it is influenced by the preservation/resection of deltoid muscle (DM), rotator cuff (RC), axillary nerve or the type of resection (intra-/extraarticular) and (3) if the preservation/resection of DM, RC, axillary nerve or the type of resection has a negative influence on the oncological outcome. We retrospectively evaluated data of 49 patients with humeral OSA. All patients underwent resection and EPR. Complication-free survival according to the ISOLS classification was estimated by a competing risk model. Functional outcome was evaluated by range of motion (ROM) in abduction and the MSTS score. Eleven patients (22%) had at least one complication. The estimated cumulative incidence for the first complication was 18% at one year, 23% at five years, and 28% at ten years, respectively. Soft tissue failure was the most common complication. ROM and MSTS scores were significantly higher in patients where DM and RC (p = 0.043/p = 0.046) and axillary nerve (p = 0.014/p = 0.021) could be preserved. Preservation of these structures had no negative influence on the surgical margins. In conclusion, EPR is a good treatment method with an acceptable complication rate. Preservation of the abductor mechanism, when possible in the setting of obtaining negative margins, provides superior functional outcome.

## Introduction

The proximal humerus is the third most common site for OSA. In general, treatment comprises neo-adjuvant chemotherapy, wide surgical resection and adjuvant chemotherapy^1,2^. Due to advances in therapy and imaging, limb salvage can be achieved in the majority of cases. Limb salvage, especially in the upper extremity, is psychologically easier to accept for patients and shows better functional results than amputation, but reconstruction remains challenging^3,4^. Several methods for reconstruction have been reported, such as endoprostheses, allografts, allograft-prosthetic composites or autografts. In case of extensive tumor spread, resection-replantation is a valuable additional alternative to amputation^5^. Debates on the preferred method are ongoing, but endoprosthetic reconstruction (EPR) is the most widely used^6–9^. Although implant survival rates seem to be higher compared to other anatomical regions, complications are common^4,7,10,11^. In order to standardize failure modes after reconstructive surgery in cancer patients, the International Society of Limb Salvage (ISOLS) classification system was developed^12,13^. Failures after EPR of the humerus have been analyzed, but without standardized criteria and not exclusively for OSA patients. Analyses of EPR failures should comprise homogenous groups of patients to allow a better understanding of incidences and reasons for complications^14^. In this context competing risk (CR) analysis, where death is included as a competing event, has been proven to add a more realistic estimation of EPR outcomes than Kaplan-Meier analysis^15^.

Due to the proximity of the neurovascular bundle to the bone, one of the main difficulties of OSA of the proximal humerus is to achieve wide resection margins and at the same time to restore function and stability. The often-required excisions of the rotator cuff (RC), the deltoid muscle (DM), as well as the axillary nerve, are mainly responsible for poor functional outcome. Controversy remains whether routine sacrifices of the DM and the axillary nerve are generally necessary to achieve clear surgical margins and so to reduce the risk of local recurrence^16,17^.

The objective of this study was to investigate the surgical and the functional outcome after resection of OSA of the proximal humerus and EPR. Therefore we aimed (1) to evaluate the risks for primary and subsequent complications after EPR according to the ISOLS classifications; (2) to evaluate the functional outcome after tumor resection and to analyze the influence of the preservation/resection of DM, RC and axillary nerve or the type of resection (intra-or extraarticular); (3) to evaluate if the preservation/resection of DM, RC and axillary nerve or the type of resection have a negative influence on the oncological outcome (surgical margins and local recurrence).

## Materials and Methods

### Patients

From 1981 to 2014, 65 patients with humeral OSA were treated at our clinic. Out of these 49 patients underwent resection and EPR, 10 patients underwent resection and replantation, four underwent resection and reconstruction with either auto- or allograft and one patient was primarily amputated. One patient with multifocal OSA did not undergo surgery. For final analysis, only the 49 patients with EPR were included. We obtained data retrospectively from our prospective tumor registry as well as from medical and radiological records. Approval of the Ethics Committee of the Medical University of Vienna was obtained prior to this investigation and the study was performed in accordance with the relevant guidelines and regulations. Informed consent was obtained from all study-participants. Table 1 summarizes demographic data.

**Table 1:** Demographic patient data.

Female n (%)	22 (45%)
Age at surgery in years (median)	17.9 (15.1/21.4)
Follow-Up in months (median)	63.8 (37.8/128.0)
**Histology n (%)**	
conventional	40 (82%)
parosteal	3 (6%)
teleangiectatic	4 (8%)
secondary	1 (2%)
high-grade surface	1 (2%)
**Implant type n (%)**	
H-HMRS	33 (67%)
Custom-made prosthesis	13 (27%)
Ceramic-prosthesis	2 (4%)
humeral MUTARS	1 (2%)
**Cementation n (%)**	
Cementless	42 (86%)
Cemented	7 (14%)
**Regression grade n (%)**	
1	3 (7%)
2	7 (17%)
3	10 (24%)
4	8 (19%)
5	12 (29%)
6	2 (5%)

Given values are median (quartiles), except where indicated otherwise. H-HMRS: the humeral Howmedica Modular Resection System (Stryker Orthopaedics, Mahwah, New Jersey, USA); MUTARS: Modular Universal Tumor And Revision System (Implantcast, Buxtehude, Germany). Regression grade 1–6, according to the Salzer-Kuntschik classification^35^. Regression grades were not available in seven patients.

### Surgical technique

All patients, except those with parosteal low grade OSA, received chemotherapy according to either the Cooperative OsteoSarcoma Study Group (COSS) regimen or the European and American Osteosarcoma Study Group (EURAMOS 1) regimen^18,19^. All operations have been performed by one of six specialized orthopedic-oncological surgeons.

In general an anterior delto-pectoral longitudinal approach was used, with excision of the biopsy track. When the tumor infiltrated, or was suspected to infiltrate, the glenohumeral joint an extra-articular resection (n = 17; 34.7%), type 5 according to the Malawer classification, was performed^20^. Here the neck of the glenoid had to be osteotomized medial to the capsular attachments. The entire capsule and the RC had to be excised. In intra-articular resections (n = 32; 65.3%), Malawer type 1, an excision of the humeral head and the associated soft-tissue was conducted by dissection of the joint capsule and RC. As much of the DM and the RC as possible were preserved. The axillary nerve was spared (n = 23; 46.9%) when tumor spread made it safely possible. The radial nerve had to be resected in two cases and was reconstructed by interposition of autologous sural nerve grafts. After distal soft-tissue dissection, the distal humeral osteotomy was conducted at least 4 cm from the distal extent of the tumor. Intraoperatively, negative surgical margins were confirmed by frozen sections.

For EPR cementless fixation of the humeral stem was performed in 42 (86%) cases with diaphyseal press-fit, the others were cemented for reasons of fixation in the conical metaphyseal bone sections.

For soft tissue reconstruction the residual DM, RC and capsule were reattached. A preservation of the deltoid muscle was possible in 22 (44.9%) patients. In 21 of these cases the distal deltoid tendon had to be released and was reattached onto the prosthesis. The Ligament Advanced Reinforcement System (LARS; JK Orthomedic Ltd, Quebec, Canada) in 14 patients, a fascia lata autograft in seven patients, and a Vicryl mesh (Ethicon, NJ, USA) in three patients were used to contain the prosthesis and to prevent subluxation.

### Follow-up

Our standard follow-up protocol included clinical and radiographic examinations of the tumor site, as well as a CT scan of the thorax and abdomen, every four months for the first three years, every six months for the following three years, and yearly thereafter^21^.

We used the ISOLS classification system to distinguish five different types of complications after EPR: In short, Type 1 represents soft tissue failures, Type 2 is aseptic loosening or non-union, respectively, Type 3 includes structural failures (e.g. implant breakage or fractures), Type 4 is infection, and Type 5 is local tumor progression^12^.

The ROM in abduction and the MSTS score^22^ were applied to evaluate functional outcomes at the most recent follow-up. For functional evaluation, patients were allocated to one of three groups of muscle resection: group I, where DM and RC could be preserved (n = 8; 16.3%), group II, where the DM, but not the RC could be preserved (n = 14; 28.5%) and group III where neither DM, nor the RC could be preserved (n = 27; 55.1%). DM was regarded preserved when more than two thirds could be spared together with the axillary nerve.

### Statistical analysis

Descriptive statistics were used to display demographic data. Statistical analysis focused on implant survival and complications after resection and EPR. We used the ISOLS criteria to classify complications. The follow-up time is described using the Inverse Kaplan Meier (KM) method and overall survival probability was estimated using the KM method^23^. Cumulative incidence of complications was estimated in a CR model, where death was modeled as a competing event. A separate CR analysis was performed for the first complication over time (irrespective of its type) and for each type of complication.

Furthermore, we evaluated differences (I) between the three afore described muscle resection types, (II) between resection/preservation of the axillary nerve and (III) intra- and extra-articular resection concerning functional outcome and local recurrence rates. We used exact non-parametric tests to compare MSTS scores and ROM with achieved resection margins between the different groups.

SPSS and SAS were used for statistical analysis.

### Ethics committee

The ethics committee has approved this study. Study number: 767/2008.

## Results

Thirteen patients (26.5%) died throughout follow-up. The overall survival (OS) rates according to KM were 91% at one year, 70% at five and 70% at ten years, respectively. Metastasis-free survival at 5 and 10 years in patients without metastasis at diagnosis was 68% and 63%, respectively.

### Surgical outcome

**Figure 1:**
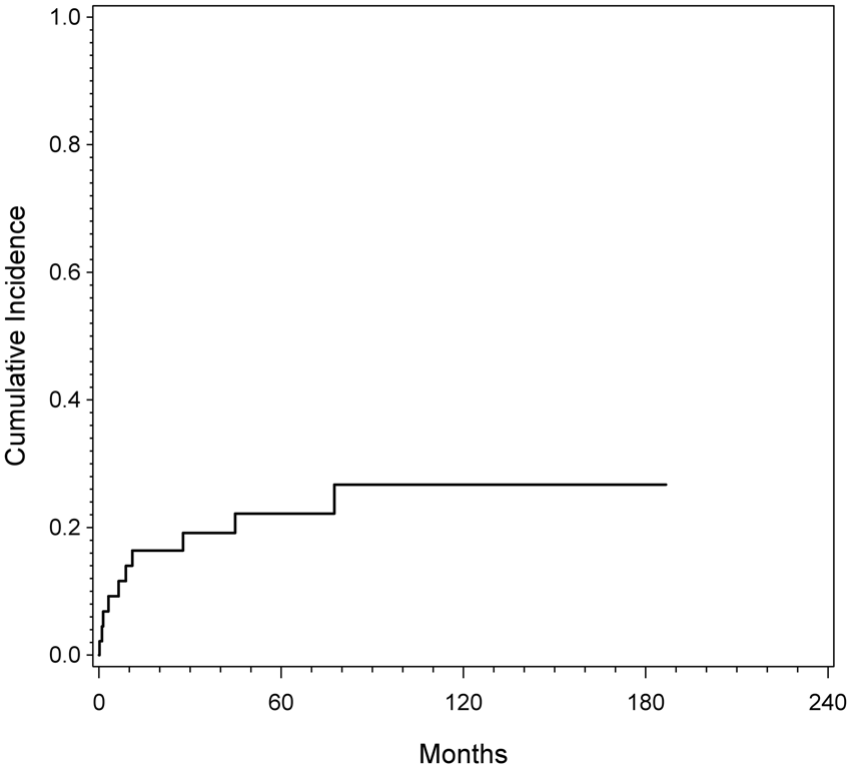
Cumulative incidence of complications estimated by CR analysis. The cumulative incidence was estimated to be 17.9% (CI 95% = 8.3% to 30.5%) after one, 23.4% (CI 95% = 11.9% to 37.2%) after five years and 27.7% (CI 95% = 14.2% to 43.1%) after ten years.

**Figure 2:**
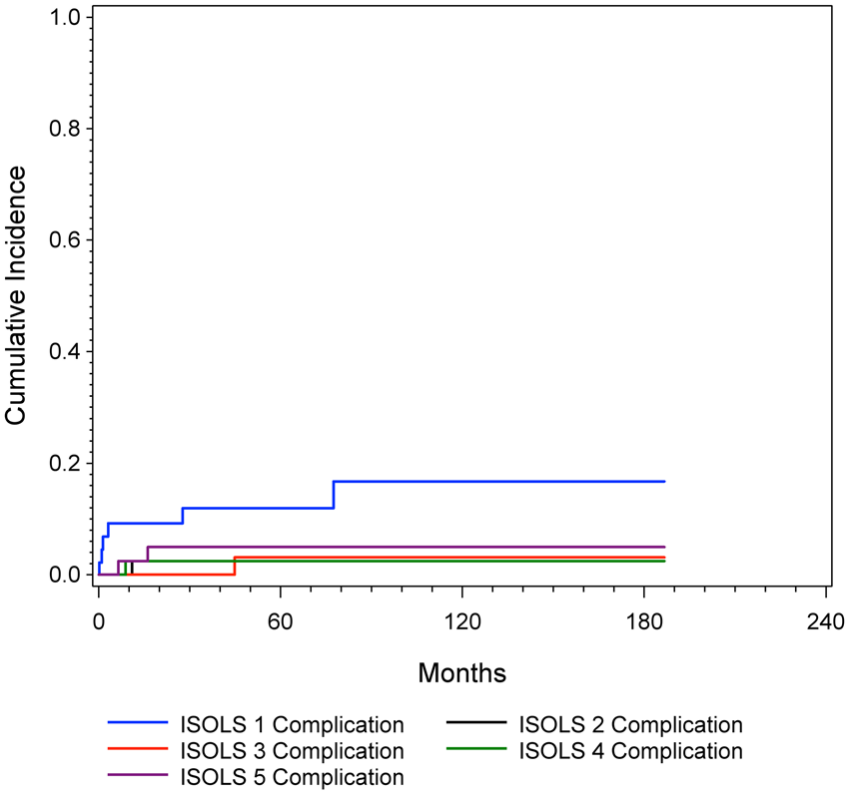
Competing risk analysis for ISOLS 1 to 5 complications. The cumulative incidence was estimated to be 8.8% (CI 95% = 2.8% to 19.3%) after one, 11.4% (CI 95% = 4.1% to 22.8%) after five years and 16.1% (CI 95% = 5.8% to 30.9%) after ten years for ISOLS 1 (soft tissue failure); 2.3% (CI 95% = 0.2% to 10.8%) after one, five and ten years for ISOLS 2 (aseptic loosening); 2.3% (CI 95% = 0.2% to 10.8%) after one year, 5.3% (CI 95% = 0.9% to 15.9%) after five and ten years for ISOLS 3 (structural failure); 2.3% (CI 95% = 0.2% to 10.8%) after one, five and ten years for ISOLS 4 (infection); 2.3% (CI 95% = 0.2% to 10.8%) after one year and 4.7% (CI 95% = 0.8% to 14.3%) after five and ten years for ISOLS 5 (tumor progression).

Overall, eleven (22.4%) patients had at least one complication and in total 12 complications were recorded. The estimated cumulative incidence for the first complication was 17.9% at one year, 23.4% at five years and 27.7% at ten years, respectively. Figure 1 depicts incidence for the first ISOLS complication (irrespective of its type). Out of 12 complications six were soft tissue failures (ISOLS type 1), one was an aseptic loosening (ISOLS type 2), two were structural failures (ISOLS type 3), one was an infection (ISOLS type 4) and two were local tumor progressions (ISOLS type 5). The latter two patients required secondary amputation, resulting in an overall limb salvage rate of 96%. Figure 2 shows the cumulative incidence for the different ISOLS types of complications.

### Functional outcome

**Figure 3:**
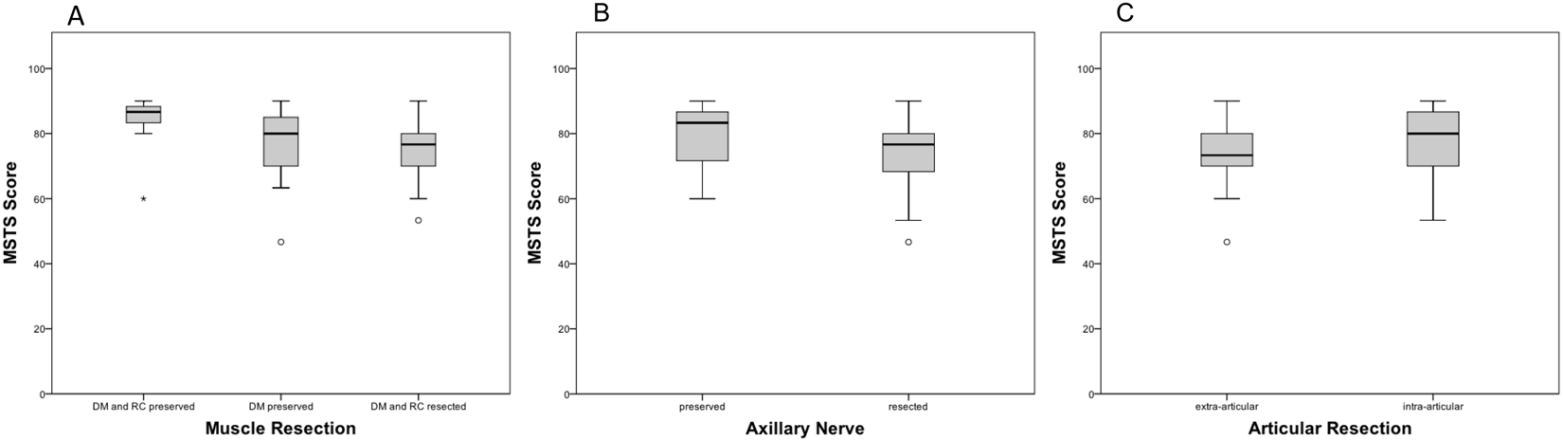
Comparison of the MSTS score between: (**A**) types of muscle resection (p = 0.046); (**B**) preservation/resection of the axillary nerve (p = 0.021); (**C**) intra- and extra-articular resection (p = 0.233).

**Figure 4:**
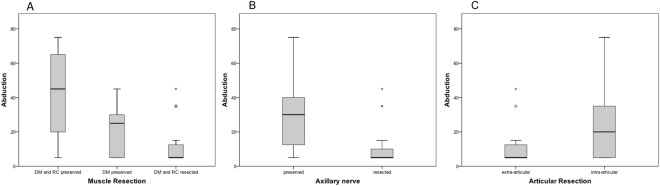
Comparison of the ROM in abduction between: (**A**) types of muscle resection (p = 0.043); (**B**) preservation/resection of the axillary nerve (p = 0.014); (**C**) intra- and extra-articular resection (p = 0.155).

Overall, patients achieved a median (25^th^/75^th^ percentile) MSTS score of 80% (70%/87%) and a ROM of 20° (5°/35°). Patients achieved significantly higher MSTS scores and greater ROM when DM and RC could be preserved (87%; 83%/88% and 45°; 20°/65°, respectively), than patients in whom only the DM could be preserved (80%; 70%/87% and 25°; 5°/30°, respectively) or patients where both muscle groups had to be sacrificed (77%; 70%/80%; p = 0.046 and 5°; 5°/13°; p = 0.0043, respectively). Patients with an intra-articular (80%; 70%/87% and 20°; 5°/35°, respectively) resection achieved higher MSTS scores and ROM than patients with extra-articular resection (73%; 70%/80% and 5°; 5°/15°, respectively), but differences did not reach significance (p = 0.233 and p = 0.155, respectively). Patients with preservation of the axillary nerve (83%; 72%/87% and 25° 0°/40°, respectively) had higher MSTS scores and ROM than patients with axillary nerve resection (77%; 67%/80%; p = 0.021 and 5°; 5°/10°; p = 0.014, respectively). Figures 3 and 4 show corresponding results.

As implants improved during the study period, we additionally divided study participants in those with early (Ceramic prosthesis and Custom-Made Prosthesis; n = 15) and those with recent implants (H-HMRS and humeral MUTARS; n = 34) and compared functional results between these two groups. Further we repeated functional outcome analysis separately between early and recent implants. In the early group the MSTS score was 73% (70%/77%) and ROM was 0°(0°/5°), respectively. In the recent group MSTS score was 80% (70%/87%) (p = 0.242) and ROM was 10° (0°/30°) (p = 0.089), respectively. In patients with early endoprosthesis we could no longer detect differences of the MSTS score and the ROM between preservation/resection of axillary nerve, extent of muscle resection or articular resection type, but the number of patients was low (data not shown). In the group with modern implants preservation of the axillary nerve still significantly improved the MSTS score (p = 0.04) and the ROM (p = 0.018). We also found a clear functional improvement when RC and DM or DM could be preserved, although differences did not reach statistical significance (p = 0.085 for MSTS score and p = 0.072 for ROM, respectively).

### Resection margins and local recurrence

A wide resection was achieved in 44 patients (90%), a marginal resection in four and an intralesional resection in one patient. This patient had a metastatic disease at diagnosis and died 5 months after surgery and palliative surgery aimed at pain relief and tumor-mass reduction. In statistical analysis neither the extent of any muscle preservation (p = 0.746) nor the preservation of the axillary nerve (p = 0.862), or the extent of articular resection (p = 0.952) showed a statistically significant influence on the resulting surgical margins. Two patients treated in the 1980ies developed local recurrence (4%). Hence, the number of local recurrences in our cohort was too low to perform statistical analysis. Both patients with local recurrence had undergone extra-articular resection, resection of the axillary nerve and resection of the DM as well as the RC for vast tumor extension. Table 2 shows a descriptive comparison of resection types and OS, local recurrence free survival and surgical margins.

**Table 2:** Descriptive comparison of articular resection type, extent of muscle resection and axillary nerve preservation/resection with overall survival (OS), local recurrence free survival (LRFS) and resection margins.

	OS		LRFS		Margins		
	5 years	10 years	1 year	5 years	wide	marginal	intralesional
**All**	70%	70%	98%	96%	90% (n = 44)	8% (n = 4)	2% (n = 1)
**Articular resection**							
intra-articular	77%	77%	100%	100%	91% (n = 29)	6% (n = 2)	3% (n = 1)
extra-articular	59%	59%	93%	85%	88% (n = 15)	12% (n = 2)	0
**Muscle resection**							
DM/RC preserved	83%	83%	100%	100%	100% (n = 8)	0	0
DM preserved	76%	76%	100%	100%	93% (n = 13)	7% (n = 1)	0
DM/RC resected	64%	64%	95%	90%	85% (n = 23)	11% (n = 3)	4% (n = 1)
**Axillary nerve**							
preserved	78%	78%	100%	100%	91% (n = 21)	9% (n = 2)	0
resected	64%	64%	95%	90%	88% (n = 23)	8% (n = 2)	4% (n = 1)

## Discussion

Complications after humeral OSA resection are common and reconstruction of the shoulder with satisfying functional results remains challenging. The current study evaluates complications in patients with humeral OSA undergoing resection and EPR, aiming to compare functional outcomes after different extents of muscle resection with regard to a potential risk for recurrence. We were able to show that: EPR is an oncological and surgical safe treatment option with an overall limb salvage rate of 96%, and an overall complication rate of 23% after five years and 27% after ten years, respectively, in a CR model. Functional outcomes are highly influenced by the extent of muscle resection and resection of the axillary nerve. Sparing these soft tissue structures was safely possible in selected cases and did not deteriorate the achievement of adequate surgical margins as a premise for local tumor control.

This study has several limitations. First, it is a retrospective analysis, therefore clinical examinations were not performed blinded and patients have not been stratified along a homogenous follow-up. Second, the time frame of the study period was relatively long. Although all patients were treated with standardized chemotherapy as well as surgical resection, additional options in diagnosis and treatment, as well as surgical techniques and implants have improved over time. In this context, we also have to mention that we included patients with parosteal OSA. These patients did not receive chemotherapy, but a wide surgical resection is also required in this subtype^21^. Since this work mainly focuses on surgical and functional outcomes, we choose to include them. Also, we aimed at evaluating functional outcomes of older versus modern prosthetic designs. Third, although, to the best of our knowledge, this is the largest series investigating the oncological, surgical and functional aspects of humeral OSA, the sample size is rather small.

We used a CR analysis to evaluate the risk of complications. In a former study, it could be demonstrated that CR analysis considerably reduces the risk estimates for all types of endoprosthetic complications in OSA patients and that these lower failure rates may better reflect reality since they account for the high competing risk of death of disease^15^. As a consequence, this might reflect a more realistic description of endoprosthetic survival in oncologic patients. To the best of our knowledge this is the first CR analysis focusing on EPR after humeral OSA.

Overall 11 patients (22%) had at least one complication according to the ISOLS classification and CR analysis revealed a risk of 18% at one year, 23% at five years and 27% at ten years. Soft tissue failures appeared in six patients and were the most common complications. Most type 1 complications were related to instability. This correlates with a study by Potter *et al*. where instability-related complications were the most common after humeral EPR and were seen in five of 16 patients (31%)^6^. Henderson *et al*. noted that type 1 complications are more likely in polyaxial joints, such as the proximal humerus and appear significantly more often in the upper extremity^13^.

Aseptic loosening was seen in one patient. The frequency was similar to other studies that reported aseptic loosening in 0 to 6%^4,24,25^. Two patients had a structural failure. In one, the prosthesis broke during a car accident, which can be regarded as a causative trauma. In the other case, a screw broke. Aseptic loosening and structural failures are less common in the upper extremity and in polyaxial joints. This can be attributed to a lesser mechanical fatigue in the non-weight-bearing upper extremity. Also torsional forces are not as high on humeral stems because one side of the stem can move freely in the glenoid^11,13^.

In general, infections are the most common complication after EPR following tumor resection (11%)^13,26^. Again, infections seem to be less common in the upper versus the lower extremity^26^. In our cohort, one patient had to undergo a revision, because of a periprosthetic infection. These results are comparable with earlier reports on EPR following tumor resection, which described periprosthetic infections in five out of 100 patients^4^ and in one out of 18 patients^27^.

Finally, local recurrence appeared in two patients in this series. Both patients who suffered local tumor progression underwent EPR at the beginning of the 1980s and were secondarily amputated. Local recurrence has been described as a common problem in tumors close to the shoulder, due to the proximity of the bone to the neurovascular bundle^4,28^. A British study reported 15 local tumor recurrences in 100 cases. However, in their cohort patients also underwent humeral resection, because of metastasis^4^. Other authors found a local recurrence rate of 13% in patients with humeral OSA^16^.

Functional results were acceptable and patient satisfaction was high, although mobility in abduction was clearly reduced. MSTS scores were 80% and results were in accordance with former studies^4,6,29^. Looking at influential factors on the functional outcome, we could show that preservation of the DM, the RC and the axillary nerve clearly improved the function. Although discussed in literature, these factors have not been proved so far^4,30^. It seems self-evident that function improves with less muscle resection, but preservation of the abductor mechanism seems to be the key for satisfying functional outcome. In our cohort preservation of the DM was possible in 22 patients and we found no local recurrence in this group. Our results go along with findings of Gupta *et al*. that in selected cases DM and axillary nerve sparing is safely possible^16^, contributing to improved functional outcome. In our study only anatomical prostheses were used. These have been criticized to function rather as a simple prosthetic spacer than a joint reconstruction^31,32^. The fact that the core design of all prostheses used in this study has not changed over time, intuitively explains the lack of differences in the functional outcomes of older versus modern prosthetic types. Over the last years, however, inverse modular prostheses, which are highly dependent on DM function, were implemented in oncological surgery and show very promising results in so far limited retrospective reports^27,33,34^. This development apparently emphasizes the importance of DM and axillary nerve preservation even further^33,34^.

With a potential impact on OS, it is an actual matter of debate, whether deltoid-sparing resection techniques have a negative influence on achieving wide resection margins and local tumor control^16,17^. Here, our data suggest that in selected cases perseveration is safely possible, as long as resection obtains clear margins. Both patients with local recurrence underwent extra-articular resection, resection of the axillary nerve and resection of the DM as well as the RC. We also did not find higher rates of marginal or intralesional resection when the axillary nerve or the DM could be preserved. We strongly believe that exact radiological diagnosis and surgical planning are able to allow intra-articular resection and sparing of a great portion of surrounding muscles in many patients. In fact, when looking at our surgical approach over time, extra-articular resections were mostly undertaken in the earlier phases of the study period, and still the only cases of local recurrence date back to this very phase. With improved diagnostic means we more frequently indicated lesser extents of resection without any further negative impact on surgical margins, and potentially OS. However, we have to point out that these results do not suggest that preservation of the axillary nerve, the DM and the RC is possible in all patients with OSA of the proximal humerus. Negative surgical margins must be achieved and the type of resection does not determine the margin; rather the desired margin determines what needs to be resected.

## Conclusion

In conclusion, EPR is a good and safe treatment option after tumor resection of OSA with an acceptable complication rate. Soft tissue failures account for most of the revisions. The preservation of the abductor mechanism seems to be the predictor of the functional outcome. Sparing these structures is safely possible in selected cases and does not deteriorate the achievement of adequate surgical margins in these patients.
